# Type IV Esophageal Atresia with Nasogastric Tube in Stomach

**DOI:** 10.21699/jns.v6i2.582

**Published:** 2017-04-15

**Authors:** Jose María Lloreda-García, Sandra Sevilla-Denia, Jose Luis Leante-Castellanos, Carmen Fuentes-Gutiérrez

**Affiliations:** Neonatal Intensive Care Unit, Hospital Universitario Santa Lucía, Cartagena, Spain

A preterm newborn required pulmonary surfactant administration and high frequency oscillatory ventilation due to respiratory distress syndrome. VACTERL association was suspected after diagnosis of several congenital anomalies (butterfly vertebra, double left ureteral system, double outlet right ventricle and limbs anomalies). A nasogastric tube was passed and tip position was confirmed in the stomach (Fig.1A). He experienced problems due to high leaks rate by endotracheal tube and progressive gastric distension, which led to esophageal atresia suspicion despite image of the tube tip into the stomach. An esophagogastric contrast study was performed to rule out tracheoesophageal fistula, showing a proximal esophageal pouch (Fig.1B). The newborn died 48 hours after birth due to hypoxemic respiratory failure. Autopsy confirmed an esophageal atresia type IV, with proximal and distal tracheoesophageal fistula. 

The passage of a nasogastric tube does not always exclude the presence of an esophageal atresia. Nasogastric tubes that passes through the larynx, the trachea and the distal fistula and reaches the stomach have been described [1-3]. In our case, nasogastric tube could reach the airway through the upper fistula and then through the inferior fistula reach to the digestive tract.

## Footnotes

**Source of Support:** None

**Conflict of Interest:** None

## Figures and Tables

**Figure 1:**
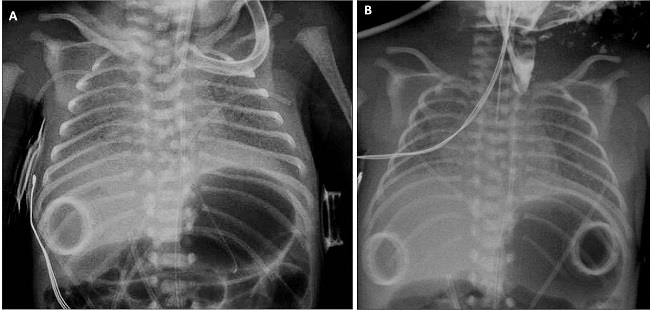
A) Nasogastric tube tip in stomach. B) Contrast study with proximal esophageal pouch.

## References

[1] King SK, Teague WJ (2016). An unusual passage of a nasogastric tube in esophageal atresia. J Pediatr.

[2] Patel RV, Jackson PB, De Coppi P, Pierro A (2013). Exclusion of oesophageal atresia by passage of a nasogastric tube: an exception to the rule. BMJ Case Rep.

[3] Soccorso G, England RJ, Godbole PP, Fisher RM, Marven SS (2012). Mind the gap: delayed diagnosis of oesophageal atresia and tracheo-oesophageal fistula due to passage of a nasogastric tube. Arch Dis Child Fetal Neonatal Ed.

